# Evaluating the Effectiveness of an Intimate Partner Violence Training Intervention on Healthcare Providers’ Preparedness, Knowledge, and Experiences: A Mixed-Methods Study From Nepal

**DOI:** 10.1177/08862605261444014

**Published:** 2026-05-19

**Authors:** Reena Koju, Rachana Shrestha, Jayanti Dhungana, Achyut Lamichhane, Amit Misra, Anna Mia Ekström, Keshab Deuba

**Affiliations:** 1 Public Health and Environment Research Centre (PERC), Lalitpur, Bagmati, Nepal; 2 Knowledge to Action (K2A), Lalitpur, Nepal; 3 Karolinska Institutet, Stockholm, Sweden; 4 Nepal Police Hospital, Kathmandu, Nepal; 5 Södersjukhuset, Stockholm, Sweden; 6 Global Health Epidemiology Group, Centre for International Health (CIH), University of Bergen, Norway

**Keywords:** Nepal, intimate partner violence, health personnel, intervention, training, readiness, knowledge and attitude, mental health

## Abstract

Intimate partner violence (IPV) places a considerable burden on health systems globally due to its profound effects on women’s health, and women who experience violence often seek care from healthcare providers (HCPs). However, HCPs often lack the preparedness and confidence to respond effectively, resulting in missed opportunities for support and care. This study, conducted in Nepal, evaluated the impact of structured training intervention on HCPs’ perceived preparedness, knowledge, and attitudes toward managing IPV and its mental health consequences, including self-harm and suicidal tendencies. The study was nested within a larger cluster randomized trial. A convergent mixed-methods design with a comparison group was conducted among 46 female HCPs in all public hospitals (except one) and 17 primary healthcare centers in Madhesh Province, Nepal. The intervention group (*n* = 24) received a 10-day intensive IPV and mental health training, while the control group (*n* = 22) completed 3-day training. Quantitative data were collected using a validated self-administered Physician’s Readiness to Manage IPV questionnaire and IPV consequences scale. Paired and independent *t*-tests were applied to assess changes. Insights from key informant interviews were thematically analyzed to explore participant experiences and perceived impacts. At baseline, over 80% of participants had not received IPV management training. Post-intervention, significant improvements were observed in HCPs perceived preparedness (median change 2.1; 95% confidence interval (CI): 1.1– 2.9), knowledge (median change 2.7; 95% CI: 2.0–3.1), and awareness of IPV consequences (mean difference 1.2; 95% CI: 0.5–2.0), with greater gains in the intervention group. Qualitative findings revealed enhanced confidence in identifying IPV, addressing psychological impacts, and supporting survivors through safety planning and referral. The training significantly improved HCPs’ knowledge, preparedness, and confidence to manage IPV and related mental health issues, underscoring the need to scale similar programs to frontline providers, particularly in rural and underserved settings, to strengthen health system’s response to IPV.

## Introduction

Intimate partner violence (IPV), a pervasive form of gender-based violence, profoundly affects the physical, mental, and social well-being of women and girls worldwide (World Health Organization & Pan American Health Organization, 2012). IPV encompasses physical, sexual, and psychological abuse, as well as controlling behaviors by intimate partners in current or past relationships. A 2021 World Health Organization (WHO) multi-country study conducted across 15 sites in 10 countries found that IPV is strongly associated with a range of both immediate and long-term adverse outcomes, such as self-reported poor health, suicidal ideation, unintended pregnancies, and abortion (Potter et al., 2021). IPV is also linked with sexually transmitted infections, chronic pelvic pain, pregnancy-related complications, homicide, and mental health disorders such as depression, anxiety, and post-traumatic stress (Martin-Engel et al., 2021; World Health Organization, 1997).

In Nepal, one in four women reports experiencing IPV, and healthcare facilities often serve as their first or only source of formal support (Ministry of Health and Population, & New Era 2022a; Clark et al., 2019; World Health Organization, 2013). Women commonly seek care for IPV-related injuries or associated conditions, offering healthcare providers (HCPs) a critical opportunity to identify and respond to IPV, even without explicit disclosure. Survivors often view HCPs as trusted professionals to whom they can disclose violence exposure (World Health Organization, 2013), yet many lack the necessary knowledge, skills, and confidence to manage IPV cases effectively, leading to missed opportunities for early detection, intervention, and referral (Deuba et al., 2024; Ministry of Health and Population & New Era, 2022b).

Training interventions have been shown to strengthen HCPs’ capacity to identify and support IPV survivors (Abeid et al., 2016; Arora et al., 2021; Mostafa Arrab & Shabaan Ibrahim, 2018). Studies from both high-income and low- and middle-income countries (LMICs) highlight persistent gaps in HCPs’ preparedness and confidence in addressing IPV (Alhalal, 2020a; Ambikile et al., 2020; Guruge et al., 2021). A global review of 19 intervention trials found that IPV-focused training significantly enhanced HCPs’ readiness including safety planning and referral practices (Kalra et al., 2021). Similarly, structured IPV training in Turkey improved HCPs’ attitudes and enhanced knowledge (Kaplan & Komurcu, 2017). Integrating IPV response into routine health services, particularly in LMICs, is essential for long-term sustainability and scale-up. Evidence indicates that such integration can enhance both survivor outcomes and health system responsiveness (Colombini et al., 2017; Oram et al., 2022).

Despite the introduction of a national clinical protocol on gender-based violence by Nepal’s Ministry of Health and Population in 2015, major gaps remain in the training, supervision, and institutional support needed to operationalize IPV management guidelines (Deuba et al., 2024; Ministry of Health and Population, 2015; Sikder et al., 2021). This challenge is particularly pronounced in Madhesh Province, which reports one of the highest IPV prevalence rates in Nepal (Ministry of Health and Population & New Era, 2022a). Strengthening HCPs’ capacity in this context is therefore critical for effective health system’s response.

To address this gap, a targeted training intervention based on cognitive behavior was implemented among female HCPs working in public health facilities in Madhesh Province to assess the impact of training on HCPs’ perceived preparedness, perceived and actual knowledge, and attitudes toward IPV management, as well as to evaluate its effectiveness in equipping providers to address the complex health and mental health consequences of IPV.

## Methodology

### Study Design

We employed a convergent mixed-methods design to evaluate a quasi-experimental training intervention. The study comprised an intervention group (Group 1) and a control group (Group 2) to assess the impact of training on the preparedness, knowledge, and attitudes of HCPs regarding the identification and management of IPV. The quantitative component consisted of pre- and post-training self-administered surveys. The qualitative component comprised key informant interviews (KIIs) with HCPs from Group 1. These interviews were designed to explore HCPs’ experiences, perceptions, and reflections specifically regarding the comprehensive training curriculum and its subsequent application to IPV care. Both qualitative and quantitative data were analyzed separately and then integrated to provide a comprehensive understanding of the findings.

### Setting

This study was conducted in Madhesh Province, Nepal, where the Nepal Demographic and Health Survey, indicates that 37% of women have experienced physical or sexual violence, and 22% of ever-partnered women experiencing physical violence perpetrated by their husband or intimate partner within the past 12 months (Ministry of Health and Population & New Era, 2022a). As part of the broader Domestic Violence Intervention (DeVI), a cluster randomized controlled trial designed to reduce IPV and related psychological distress, our training evaluation was implemented prior to the main DeVI intervention. Details of the DeVI trial design have been described elsewhere (Shrestha et al., 2023) . This study is a nested sub-study within the DeVI trial. Thus, it did not involve additional facility-level randomization. Twenty-four public health facilities (all public hospitals except one, and 17 randomly selected primary healthcare centers [PHCCs]) participated and randomized at the facility level to Group 1 (12 intervention sites) or Group 2 (12 control sites) using a computer-generated random number table in the ratio of 1:1.

### Participants

This study was nested within a larger trial that utilized a facility level cluster randomization approach. The initial sampling frame consisted of 43 healthcare facilities in Madhesh Province, identified based on the volume of female clients seeking health services. Facilities were randomized in a 1:1 ratio into intervention and control groups using a computer generated random number table. The randomization process concluded once the predefined requirement for clusters was achieved. Within the selected health facilities, HCPs were recruited based on predefined inclusion criteria of working in the maternity department, involved in DeVI trial and those that are in frequent contact with women experiencing violence. While selecting the HCPs, no discretion was allowed at the facility level to minimize the selection bias. We initially targeted 48 HCPs (24 from Group 1 sites and 24 from Group 2 sites). A total of 46 HCPs completed the initial training and assessments (Group 1: *n* = 24; Group 2: *n* = 22). We prioritized nursing staff (Auxiliary Nursing Midwives [ANMs], senior ANMs, and nurses), given their frontline role in maternity services and frequent contact with women who may experience IPV. One psychosocial counselor also took part in the training. Evidence suggests that nurses can significantly enhance IPV screening and survivor care (Eapen & Santa Maria, 2024).

Subsequent training batches were organized due to attrition (staff transfers, further education, or maternity leave), resulting in 63 HCPs trained across 24 facilities (33 from Group 1 and 30 from Group 2). Analyses were restricted to the original cohort of 46 HCPs because complete pre- and post-training assessments were not available for later batches.

### Training Duration

Training was delivered in Bardibas, Mahottari District, Madhesh Province, from May 26 to June 4, 2022. Group 1 received an intensive 10-day psychosocial counseling-oriented IPV training (8 hr per day). Group 2 received a 3-day training course (8 hr per day). HCPs were given daily subsistence allowance for all training days. Because it was a residential training, lodging and meals were covered by the study team.

### Training Intervention: Structure, Content, and Delivery

The IPV training curriculum was designed to enhance HCPs’ capacity to identify, counsel, and refer women encountering IPV. The training curriculum was adapted from the World Health Organization’s Problem Management Plus (PM+; World Health Organization, 2018), which is an evidence based psychological intervention designed for implementation by non-specialists in low-resource settings. To address the specific complexities of IPV, the core PM+ competencies were integrated with tailored modules focusing on safety planning (developing a safety plan, identifying a safe place to be in case of danger, having the legal and other important documents in place, developing a code word, keeping important numbers in a mobile phone) and empowerment-based components such as discussion about self-esteem and coping strategies (British Columbia, 2015; FORGE, 2013). This adapted model was designed to equip HCPs with the skills to address both the psychological distress and the immediate safety requirements of survivors. It integrated foundational knowledge with practical psychosocial competencies. A formative assessment was performed with key stakeholders, including women with lived experiences of abuse, local HCPs, community-based groups, and representatives from federal and province levels to ensure cultural and contextual relevance. The draft curriculum was subsequently reviewed and finalized through several rounds of consultations with experts in IPV and mental health.

The final training design aimed to enhance HCPs’ knowledge, attitudes, and preparedness for managing IPV and its related mental health consequences. Group 1 (intervention group) participants underwent a 10-day intensive training that included practical skill-building sessions, whereas Group 2 (control group) participated in a 3-day foundational program addressing basics of essential IPV and mental health principles. Table 1 summarizes the topic content addressed in both training modalities.

**Table 1.** Summary of Contents of the Training Across Groups.

Core training modules (Both Groups)	Group 1 only
a. Introduction to IPV and domestic violence (DV): types of violence, causes, and consequences for women, children, and society; common health consequences of IPV/DV b. Global, national, and provincial epidemiology of IPV/DV c. Introduction to mental health: causes, effects, stigma, and psychosocial impact on women and families; stigma and mental health d. Epidemiology of mental health problems at global, national, and provincial levels e. Relationship between violence and mental health f. Social support: types, importance, and methods to strengthen support systems • Ethical principles in research and clinical practice: informed consent, privacy, and confidentiality • Assisted referral for participants with severe mental health problems or recurrent IPV exposure	a. Role of healthcare settings in addressing IPV in Nepal; emotional-support counseling and motivational interviewing techniques b. Integrated multi-component IPV and mental health intervention; understanding adversity and cycles of violence c. Psychoeducation on IPV (cyclical nature of abuse, power and control, gender roles, forms of violence, consequences) and mental health d. Risk assessment: identifying IPV victims, asking sensitive questions, and responding effectively e. Helping skills: building rapport, active listening, and therapeutic communication f. Emotion regulation and stress management (breathing techniques, behavioral activation, relaxation methods) g. Problem management: steps of managing problem, distinguishing solvable and unsolvable problems; creating problem solving plans h. Safety planning: developing and monitoring safety plans, identifying barriers and facilitators. i. Managing challenging clients and maintaining professional boundaries. j. Enhancing social support: leveraging formal and informal networks, community linkages

Participants in Group 1 were specifically trained on practical strategies, including stress management techniques, problem-solving approaches, safety planning, and addressing barriers to safety behavior (British Columbia, 2015; Goodman et al., 2003; Walker, 1979; World Health Organization, 2018). These modules were designed to equip HCPs with practical tools for supporting IPV survivors. The HCPs were blinded to their group assignment, whereas the trainers were aware of the allocation. Although participants naturally became aware of the training content during the sessions, this did not disclose their group assignment.

HCPs were provided with training manuals, referral brochures, and supplementary materials for future use. All materials were reviewed and approved by experts in violence prevention, mental health, and public health prior to implementation, as detailed in prior publication (Shrestha et al., 2023).

### Mode of Training

**Figure 1.**
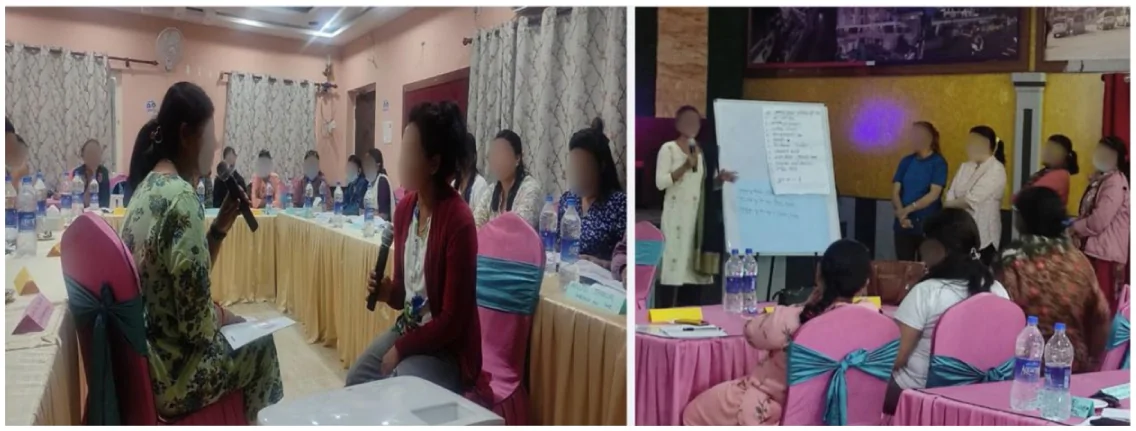
Role-play and group discussion activities during the training.

A multidisciplinary Nepali-speaking team comprising psychiatrists, psychologists, psychosocial counselors, violence prevention specialists, public health experts, and study team members delivered the training. The training content was delivered through interactive and participatory learning methods to encourage peer learning. Each session was delivered in combination with lectures and small-group discussions, vignettes, reflective exercises, case studies based on formative research conducted in Madhesh Province (Figure 1). Interactive activities and recreational elements were incorporated to enhance participant engagement. Role-plays were conducted in paired or triad format of counselor*–*client*–*observer, allowing HCPs to practice counseling techniques. The facilitators also demonstrated appropriate counseling behaviors prior to the mock sessions of HCPs. Role-plays by HCPs were evaluated using pre-specified criteria focusing on privacy and confidentiality, appropriate non-verbal communication, and overall intervention fidelity. The training did not anticipate major adverse events. However, one participant who related to the case examples in the manual became sad and experienced heightened stress. She was counseled by the psychiatrist, who was also one of the trainers.

*Left*: Participants engaged in role-play exercises simulating patient–provider interactions during the training sessions. *Right*: Participants presenting outcomes of their group discussion as part of collaborative learning activities.

### Outcome Measures

Primary outcomes were HCPs’ IPV-related knowledge and preparedness (Table 2) to identify and manage IPV. A secondary outcome was change in HCPs’ attitudes toward women experiencing IPV. A nine-item scale assessed knowledge of IPV consequences (Table 2).

**Table 2.** Overview of the Instruments Used in the Study.

S.N.	Tool/domain	Number of items	Examples of questions
1.	PREMIS		
1.1.	Perceived preparedness	8	How prepared you feel to: a. Ask appropriate questions about IPV b. Appropriately respond to disclosures of abuse c. Help an IPV victim create a safety plan
1.2.	Perceived knowledge	10	How much do you feel you know about: a. Signs or symptoms of IPV b. Perpetrators of IPV c. Referral sources for IPV victims
1.3.	Actual knowledge	19	a. What is the strongest single risk factor for becoming a victim of IPV? b. Which of the following is the most appropriate way to ask about IPV?
1.4.	Attitude	19	a. If an IPV victim does not acknowledge the abuse, there is very little that I can do to help b. I ask all new patients about abuse in their relationships
1.5.	Practice	10	a. How many new diagnoses of IPV would you estimate you have made in last 6 months? b. In the past 6 months, which of the following actions have you taken when you identified IPV?
2.	IPV consequences (World Health Organization, 2013)	9	Which of the following are the consequences of IPV? (injuries, repeated pregnancy, headache, etc.)

The qualitative component explored HCPs’ experiences and reflections on the training, emphasizing changes in their understanding of IPV, confidence in case management, and the perceived relevance of newly acquired skills in clinical practice, while also addressing contextual factors that affected their capacity to incorporate IPV response into routine care.

### Data Collection Tools and Variables

Quantitative data were collected using pre- and post-training self-administered questionnaires based on a modified version of the Physician’s Readiness to Manage Intimate Partner Violence Survey (PREMIS; Deuba et al., 2024; Short, 2006). Questionnaire sections included respondents’ demographics and background, perceived preparedness, perceived knowledge, actual knowledge, attitudes/opinions, and practice-related items.

The qualitative component employed a semi-structured interview guide to explore participants’ experiences, perceptions and reflections on the training. KIIs were conducted with Group 1 participants to gain an in-depth understanding of their perspectives.

### Data Collection Procedures

Quantitative pre-test surveys were self-administered immediately before training and post-test surveys were self-administered immediately after completion of training. Written informed consent form was obtained from the HCPs for their training participation, administration of surveys, and KIIs. It took approximately 25 to 30 min to complete the surveys. Tools adapted from PREMIS were developed with subject-matter expert input, translated into Nepali, and reviewed by a language expert for cultural appropriateness and clarity before administration.

Qualitative data were collected via face-to-face interviews that were audio-recorded. We conducted KIIs with HCPs in Group 1 using a semi-structured interview guide. These interviews took place immediately following the training, completion of the post-test survey. Written informed consent was obtained prior to interview. The study team developed the interview guide based on the study objectives and a review of relevant literature. The tool comprised open-ended questions designed to capture HCPs’ experiences with the IPV training, as well as their perceptions of its impact on their knowledge and attitudes. The qualitative interviews lasted between 20 and 25 minutes. In order to address social desirability bias, HCPs were assured that their responses would be kept confidential and used solely for the research purpose and not for the job evaluation. For the qualitative component, a total of 15 out of 24 HCPs from Group 1 were interviewed in Nepali after the post-training survey was completed by an independent study team member who was not involved in the training delivery. HCPs were interviewed by ensuring diverse representativeness, such as HCPs working at hospitals versus PHCCs and health facilities located in urban versus rural municipalities.

All collected data were stored on secure, password protected device accessible only to the core study team. To ensure participant confidentiality and anonymity, all personal identifiers were removed from the quantitative dataset and qualitative transcripts prior to final data analysis.

### Data Management and Analysis

#### Quantitative Data Management and Analysis

**Figure 2.**
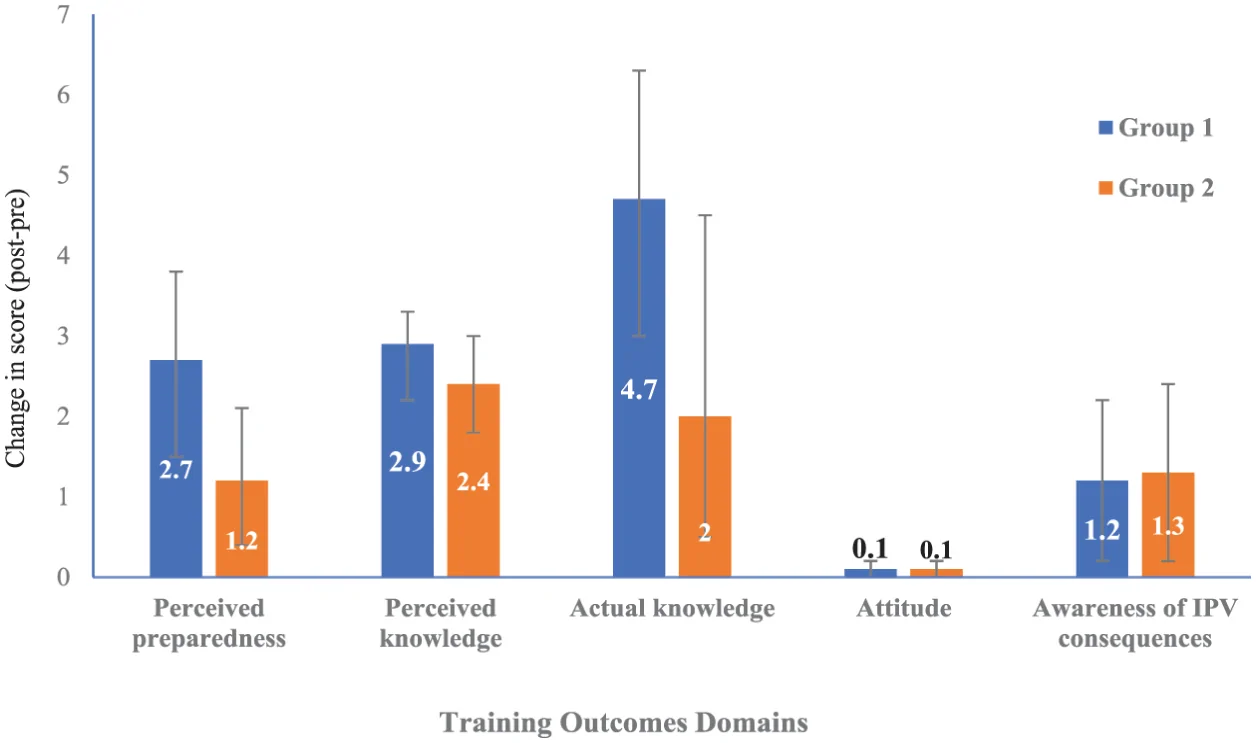
Pre- and post-training score changes across key IPV domains, by training group. *Note.* The whisker in the bar represents the confidence interval of score in each outcome.

Survey data were coded and entered into EpiData, cleaned in Excel, and analyzed in IBM SPSS Statistics for Windows, version 21.0 (IBM Corp, Armonk, NY, USA). We summarized sociodemographic characteristics using descriptive statistics. PREMIS domains were scored as per developer’s guidelines (Short, 2006). For perceived preparedness and knowledge, each HCP’s item responses were averaged to generate individual domain scores, which were then summarized by group. Actual knowledge items were scored 1 for correct and 0 for incorrect; multiple-response items awarded 1 point per correct option. Individual scores were summed and averaged by group. Attitude items were evaluated using a 7-point Likert scale, ranging from 1 (*strongly disagree*) to 7 (*strongly agree*). Scores for negatively phrased items were reverse coded prior to analysis. We then computed individual mean attitude scores, which were subsequently summarized by group. We did not administer the practice session at post-test, since the training was residential and we did not expect changes in practice during this period. IPV consequences knowledge was summed across nine items (range 0–9). Prior to the analysis, the normality of the obtained data was assessed using Shapiro–Wilk test (*n* < 50). To evaluate within-group pre- and post-training changes, we utilized paired *t*-tests for normally distributed data or Wilcoxon signed-rank tests for non-normally distributed data. The specific results of these within-group analyses are presented in Supplemental Table S1 and visually illustrated in Figure 2. Between-group post-test comparisons used independent *t*-tests or Mann–Whitney *U-*tests (Gliner et al., 2017). Two-sided *p*-value ≤ .05 was considered statistically significant.

#### Qualitative Data Management and Analysis

Qualitative interviews were conducted in Nepali, transcribed in Nepali, and then translated into English. Team members who were not involved in interviews performed transcription and translation. The transcript was then reviewed by the interviewer to ensure translation accuracy. The finalized transcript was then used for inductive thematic analysis (Braun & Clarke, 2006). For the analysis, team members who translated the interviews iteratively read the transcripts and independently developed initial codes related to the study objectives (e.g., confidence, addressing IPV, practical sessions, and stress management) in the Taguette software (Rampin & Rampin, 2021). Codes were organized into candidate themes that were refined through discussion. Themes included HCP roles in IPV identification and response, capacity building, the IPV and mental health nexus, and the perceived importance of IPV training. Representative de-identified quotations were selected to illustrate each theme.

The mental health component of the training was analyzed separately and is not reported in this manuscript due to different follow-up periods. This article focuses exclusively on the effectiveness of the IPV training component.

## Results

### Participant Background Characteristics

Table 3 presents the background characteristics of the HCPs who participated in the study. The mean age of the participants was 34.4 years (standard deviation: 7.3), and mean age was similar in both groups. Approximately 39% of the participants were aged 21 to 30 years and 39% were 31 to 40 years. Most participants (83%) were married. In terms of professional cadre, one-third were nurses, 28% were senior ANMs, and 37% were ANMs. Among them, majority of the nurses belonged to Group 1 than Group 2 (12 vs. 3).

**Table 3.** Sociodemographic Characteristics of HCPs (*N* = 46).

Variables	Group 1		Group 2		Total *n* (%)
	Hospital *n* (%)	PHCC *n* (%)	Hospital *n* (%)	PHCC *n* (%)	
Age (x̄ ± σ)	34.33 ± 7.28		34.45 ± 7.47		34.39 ± 7.29
Age distribution					
21–30	6 (13)	2 (4)	1 (2)	9 (20)	18 (39)
31–40	7 (15)	5 (11)	1 (2)	5 (11)	18 (39)
41–50	3 (7)	1 (2)	0	6 (13)	10 (22)
Marital status					
Married	11 (24)	6 (13)	2 (4)	19 (41)	38 (83)
Unmarried	5 (11)	2 (4)	0	1 (2)	8 (17)
Profession within health facility					
Auxiliary nursing midwives (ANMs)	4 (9)	3 (7)	1 (2)	9 (20)	17 (37)
Nurse	8 (17)	4 (9)	1 (2)	2 (4)	15 (33)
Senior ANMs	3 (7)	1 (2)	0	9 (20)	13 (28)
Psychosocial counselor	1 (2)	0	0	0	1 (2)
Level of education					
Proficiency certificate level in nursing	5 (11)	4 (9)	1 (2)	7 (15)	17 (37)
ANMs	5 (11)	4 (9)	1 (2)	9 (20)	19 (41)
Bachelors	5 (11)	0	0	3 (7)	8 (17)
Masters and above	1 (2)	0	0	1 (2)	2 (4)
Duration of working in current health facility (in years)					
<1	0	0	0	3 (7)	3 (7)
1–5	8 (17)	5 (11)	2 (4)	10 (22)	25 (54)
>5	8 (17)	3 (7)	0	7 (15)	18 (39)
Number of patients attended per week in maternal and child health unit of health facility					
≤50	13 (28)	2 (4)	2 (4)	8 (17)	25 (54)
>50	3 (7)	6 (13)	0	12 (26)	21 (46)
Received any training related to violence management					
Yes	7 (15)	1 (2)	0	0	8 (17)
No	9 (20)	7 (15)	2 (4)	20 (43)	38 (83)

*Note.* x̄ stands for Mean and σ for standard deviation.

About 41% of HCPs held an ANM qualification, while 37% possessed a proficiency certificate in nursing. More than half (54%) had worked at their current health facility for 1 to 5 years, and 39% for more than 5 years. Regarding service volume, 54% attended 50 or fewer patients per week, and the remaining 46% saw more than 50 patients weekly.

The majority (83%) of HCPs had not received prior training related to violence management. None of the participants in Group 2 (comparison group) had such training, whereas eight HCPs in Group 1 had previously attended short courses on gender-based violence or psychosocial counseling. KIIs corroborated that for most participants, this was their first formal training specifically focused on IPV management.

### Impact of Training on Knowledge, Preparedness, and Attitudes

The training intervention led to significant improvement in perceived preparedness, perceived knowledge, actual knowledge, and knowledge of IPV consequences among participants (Figure 2; Supplemental Table 1). Statistically significant pre- to post-changes were observed across all subscales in both groups, except for actual knowledge and attitude in Group 2.

Group 1 demonstrated a median increase of 2.7 points (95% confidence interval (CI): 1.5–3.8) in perceived preparedness, compared to a 1.2-point gain (95% CI: 0.4–2.1) in Group 2. Similarly, perceived knowledge increased substantially in both groups, with Group 1 gaining 2.9 points (95% CI: 2.2–3.3) and Group 2 by 2.4 points (95% CI: 1.8–3.0). Actual knowledge scores improved notably: Group 1 increased by 4.7 points (95% CI: 3.0–6.3) and Group 2 by 2.0 points (95% CI: 0.5–4.5). Attitude changes were minimal in both groups (mean difference = 0.1; 95% CI: 0.0–0.2). For knowledge of IPV consequences, Group 1 improved by 1.2 points (95% CI: 0.2–2.2) and Group 2 by 1.3 points (95% CI: 0.2–2.4).

To complement quantitative results, qualitative findings were derived from KIIs with HCPs in Group 1 (those who received the 10-day comprehensive training). Interviews explored HCPs’ experiences, perceptions, and reflections on the training. Themes emphasized enhanced awareness of IPV, improved confidence and counseling skills, recognition of HCPs’ roles in addressing IPV, and advocacy for wider dissemination of the program.

### Importance of Training in Enhancing Awareness and Preparedness

Before the training, most HCPs understood violence mainly as physical abuse. Afterward, they described a broadened awareness that included psychological, social, and systemic dimensions of IPV. HCPs reported the training clarified their professional responsibilities to address IPV and equipped them to respond appropriately.

Before this training, I didn’t know much about intimate partner violence. But after this training, I have now also found out about the management of such violence. (Nurse, Hospital)

We have understood that violence does not only mean physical violence; there is psychological violence and many other forms. (Nurse, PHCC)

This training has made us aware about our responsibility. To be honest, this was also one of our responsibilities, and I realized this was missing while delivering the services. (Nurse, Hospital)

Prior to this training, I was aware that violence is prevalent in every household. However, I was not aware about different stages of violence and the appropriate actions to take. (ANM, Hospital)

### Improved Confidence and Counseling Skills

HCPs consistently reported increased confidence in approaching and counseling women experiencing IPV. Many stated that they previously lacked the skills to provide psychosocial support but gained practical counseling techniques during the training.

We didn’t know how to approach for counseling and even we didn’t know the techniques of counseling. Through this training we have learnt many things about managing the mental health issues of violence survivors. (ANM, Hospital)

A confidence level has been built up to tackle if any women experiencing violence come to us. (Nurse, PHCC)

### Role of HCPs in Identifying and Addressing IPV

Participants recognized their frontline role in early detection and intervention. They emphasized establishing trust and maintaining confidentiality and creating a safe environment for disclosures are essential elements that are also evident in quantitative gains in preparedness.

Most women experiencing violence will try to meet female healthcare providers since a woman can understand another woman’s problems better. (Nurse, PHCC)

Not all women can speak openly about their household matters. This training should be given to more healthcare providers to help identify such issues. (Nurse, Hospital)

Through this training, we have discovered appropriate facilities we can use to solve problems beyond our capacity. (Nurse, Hospital)

If health care providers do not give proper attention, women experiencing violence might attempt suicide. So, if we identify such women early, we can provide counseling and prevent suicide. (Nurse, Hospital)

### Importance of Practical Sessions

HCPs considered sessions of the training as effective and practical for supporting survivors, they further emphasized sessions on problem management and safety planning as the most useful and directly applicable to women experiencing violence.

Session about problem management where the client will be encouraged that we are there to guide her for solving problem and client will give her best effort to solve the problem. (Nurse, Hospital)

Session about the development of safety plan; in this, women can develop safety plan for their security and feel safe as well. (Nurse, Hospital)

### Link Between IPV and Mental Health Challenges

HCPs described a strong connection between IPV and mental health outcomes, including chronic stress and suicidal ideation. They valued learning about early stress management and psychosocial support, which paralleled quantitative improvements in knowledge of IPV consequences.

Women go through severe stress due to experience of violence. Some even reach the stage of suicidal tendency. We can try to reduce their stress levels. (Nurse, PHCC)

If we manage stress early, there will be no space for long-term mental health problems. (Nurse, PHCC)

We have gained valuable insights into the mental health impact of IPV, including victims contemplating or attempting to end their life. (Nurse, Hospital)

This training can help prevent suicide attempts among violence survivors. (Psychosocial Counselor, Hospital)

Minor things will affect the mental health of the violence survivors and if we don’t support them, they might attempt suicide. (Nurse, Hospital)

### Post-Training Comparison Between Groups

Table 4 summarizes post-training comparisons across PREMIS subscales and knowledge of IPV consequences. For non-normally distributed variables (perceived preparedness and perceived knowledge), Mann–Whitney *U*-tests were used. During pre-test, there were no significant differences in knowledge and attitude scores between groups (*p* > .1), confirming comparability. However, in post-test Group 1 scored significantly higher than Group 2 in perceived preparedness and perceived knowledge. The median perceived preparedness score in Group 1 was 6.2 (interquartile range [IQR]: 4.0–7.0) versus 3.8 (range: 2.7–6.3) in Group 2 (*U* = 64.5, *p* < .001, *r* = .28), indicating a small but meaningful effect. The median perceived knowledge score was 6.0 (3.8–7.0) for Group 1 and 4.9 (3.1–7.0) for Group 2 (*U* = 159.5, *p* = .021, *r* = .33), representing a moderate effect.

**Table 4.** Post-Training Comparison of PREMIS Subscales and Knowledge of IPV Consequences Between Groups.

Domains	Group 1 (*n* = 24)	Group 2 (*n* = 22)	Mann–Whitney *U-*test			
	Median (min/max)	Median (min/max)	*U*	*Z*	Effect size (*r*)	*p*-value
Perceived preparedness						
Pre-test	3.3 (1.1/7.0)	2.5 (1.1/6.2)	223.0	0.9	.13	.367
Post-test	6.2 (4.0/7.0)	3.8 (2.7/6.3)	64.5	4.3	.28	.000
Perceived knowledge						
Pre-test	2.8 (1.6/6.9)	2.3(1.1/4.2)	202.5	1.3	.19	.176
Post-test	6.0 (3.8/7.0)	4.9 (3.1/7.0)	159.5	2.3	.33	.021
Actual knowledge*						
Pre-test	13.83 ± 4.99	13.00 ± 5.22	—	—	—	.583
Post-test	18.54 ± 4.23	15.00 ± 3.32	—	—	—	.003
Attitude*						
Pre-test	2.58 ± 0.18	2.76 ± 0.16	—	—	—	.516
Post-test	2.62 ± 0.15	2.72 ± 0.19	—	—	—	.443
Consequences of IPV*						
Pre-test	6.50 ± 2.22	5.23 ± 2.39	—	—	—	.068
Post-test	7.71 ± 1.27	6.50 ± 0.85	—	—	—	.001

* The variables were analyzed using independent sample *t*-test; *U* = The *U* statistic from the test; *Z* = standardized *Z*-score.

For normally distributed variables (actual knowledge, attitudes, and IPV-consequences knowledge), independent *t*-tests were conducted. Group 1 achieved significantly higher post-training scores in actual knowledge (mean 18.54 vs. 15.00; *p* = .003) and in knowledge of IPV consequences (mean 7.71 vs. 6.50; *p* = .001) compared to Group 2. Attitude scores remained comparable between groups (2.7 vs. 2.7; *p* = .443).

Qualitative interviews with HCPs also reported the need for expansion of such program to other health facilities.

### Advocacy and Empowerment

HCPs advocated for scaling the training to other health sectors and community workers. They viewed counseling as a pathway to empower women to recognize abuse, plan for safety, and access services.

It would have been better if this training was given to all healthcare providers. If everyone has the knowledge and skills, we can minimize IPV cases. (Nurse, PHCC)

I think this type of training is helpful for us especially the nursing staffs. All of the nurses of PHCCs, health posts and provincial hospitals who are involved in providing facilities in the OPD (outpatient department) need this type of training. (Nurse, Hospital)

This training is useful not only for HCPs but also for individuals in different fields such as schools and banks. (Nurse, PHCC)

### Practical Application of Skills and Knowledge

Participants highly valued the practical sessions, particularly modules on problem management, safety behavior planning, and psychosocial support techniques. These were regarded as the most applicable components for real clinical encounters. Several HCPs noted that they previously did not prioritize confidentiality or ask sensitive questions but now understood these as essential elements of survivor centered care.

The session on safety behavior planning was particularly useful. Women can plan ahead if they feel they are at risk of violence. (ANM, Hospital)

This training has taught us the step-by-step process of addressing women’s problems and managing their cases effectively. (Nurse, Hospital)

We used to encounter many women with similar cases but did not prioritize confidentiality or ask appropriate questions. Now we recognize its importance. (ANM, Hospital)

## Discussion

This study evaluated the effectiveness of a targeted training program in improving HCPs’ perceived preparedness, knowledge, and attitudes toward addressing IPV in Nepal. Before the intervention, very few HCPs had received any IPV-related training. Post-training results showed significant improvements in perceived preparedness, perceived knowledge, actual knowledge, and understanding of IPV consequences among HCPs who received enhanced training. Similar improvements have been reported from Canada and the United States (The EDUCATE Investigators, 2018), Tanzania (Abeid et al., 2016), Saudi Arabia (Alhalal, 2020b), and among dental students from United States showing significant impact across all PREMIS subscales post-intervention (McAndrew et al., 2014).

Qualitative findings indicated that HCPs felt more capable of identifying women experiencing violence, asking appropriate questions, and making necessary referrals after training. Participants also gained greater awareness of psychological, social, and systemic forms of violence, enhancing survivor-centered care. Comparable results were observed at a Canadian fracture clinic, where HCPs were more likely to incorporate IPV screening into routine care (Sprague, 2019). Targeted training for North American orthopedic surgeons, surgical trainees, and non-physician HCPs significantly improved their readiness to manage IPV cases (The EDUCATE Investigators, 2018).

Our participants recommended extending such training to all health departments, in particular emergency and outpatient care, PHCCs, and health posts, to reach a wider community, to reach women who first seek treatment at lower-level facilities for IPV-related injuries. This aligns with North American research, emphasizing the importance of training frontline providers to improve early detection and response to IPV (Etherington et al., 2021). Expanding IPV training to different departments or beyond low-level health facilities could ensure earlier identification and better management of IPV cases, thereby improving outcomes for survivors.

Although the enhanced training intervention led to significant improvements, not all between-group differences were statistically significant. Consistent with studies in Greece (Papadakaki et al., 2013), perceived preparedness and knowledge post-training improved while attitude changes were more modest (Alhalal, 2020b). According to the realistic evaluation framework proposed by Pawson and Tilley (1997), outcomes are contingent upon conducive institutional and social environments that stimulate reflection and value-driven transformation that mainly may evolve over time as mechanisms of practice and reflection are established (Pawson & Tilley, 1997). Evidence from LMICs underscores the importance of integrating IPV training into existing health systems and supported it with robust institutional frameworks (Colombini et al., 2008; Martin-Engel et al., 2021; Oram et al., 2022).

In Nepal, gender-based violence is primarily addressed by One-Stop Crisis Management Centers (OCMCs), offering comprehensive services, legal aid, medical treatment, protection, and psychosocial counseling, but their hospital-based location limits access for individuals in rural or lower-level health facilities.

Strengthening capacity of HCPs in PHCCs and health post through short, targeted training programs could enable these facilities to act as referral points for IPV survivors. Evidence suggests that even the existing OCMCs are not fully equipped to provide optimal care, underscoring the need for regular monitoring, supervision, and capacity building to ensure quality services for survivors (Deuba et al., 2024). Integrating IPV management training into the continuing professional development of frontline HCPs may strengthen the overall referral and response system, especially in underserved areas. Our findings suggest that the training strengthened HCPs confidence in addressing psychosocial distress and in linking survivors to appropriate mental health or protection services. These improvements may support suicide prevention efforts through earlier detection and referral, although direct effects on suicidal behavior cannot be inferred from this study.

This study has several strengths. Including a comparison group enhanced the internal validity and the self-administered pre- and post-training assessments provided a comprehensive evaluation of the intervention’s impact, while maintaining trainer consistency across groups minimizing implementation bias. Practical teaching methods such as role-playing, case-based discussions, and demonstrations enriched the learning experience and facilitated skill acquisition. The inclusion of HCPs from nearly all public hospitals in Madhesh Province increases the generalizability of the findings to other public facilities across Nepal. The use of validated tools, for example, PREMIS, further strengthens the reliability of the results.

Our findings indicate that while the 10-day intensive training curriculum yielded superior outcomes, the improvements observed among participants in the 3-day basic course suggest that shorter training models may offer a more scalable and pragmatic approach for resource-limited setting. Such foundational training could serve as an essential first step in building primary care capacity for trauma-informed care in areas or regions where longer training is logistically or financially prohibitive. However, it remains unclear whether a 3-day training program provides sufficient depth to sustain the complex clinical competencies required for the long-term management of IPV and its associated mental health consequences.

### Limitations of the Study

Limitations include a small, all-female sample, which limits generalizability of the findings. However, this reflects the reality that nurses and ANMs provide most maternal and child health services in public health facilities in Nepal. Self-administered questionnaires may not fully capture behavioral change in actual clinical settings and the residential format precluded assessment of clinical application. Long-term sustainability was not evaluated here, but should be part of future studies including competency-based assessments (Pedersen et al., 2021) in real-world practice. Moreover, the small sample size resulted in unadjusted comparisons.

While we did not evaluate whether these changes led to better health outcomes for women experiencing violence, practical application of these skills in clinical environments and their impact on survivor well-being should be a future focus. Incorporating role-play evaluations and direct observation of clinical practice could provide a more accurate measure of skill retention and application.

Another limitation is that the qualitative interviews were performed only with the Group 1 HCPs. However, interviewing HCPs in the Group 2 may have provided additional comparative perspectives regarding adequacy and value of shorter training formats. Although baseline characteristics were comparable between the study arms, the small sample size precluded multivariable analysis to adjust for potential confounding related to individual participant backgrounds and professional licensure.

## Conclusion

Targeted, interactive IPV training significantly improved preparedness, knowledge, and confidence among Nepalese HCPs in managing IPV and its associated consequences. Scaling up such programs across emergency, outpatient, and primary care settings could enhance early IPV identification, management, and referral, especially in rural areas. Strengthening HCP capacity throughout the health system is essential to mitigate IPV’s health consequences and improve survivor safety and well-being.

## Supplemental Material

sj-docx-1-jiv-10.1177_08862605261444014 – Supplemental material for Evaluating the Effectiveness of an Intimate Partner Violence Training Intervention on Healthcare Providers’ Preparedness, Knowledge, and Experiences: A Mixed-Methods Study From Nepal Supplemental material, sj-docx-1-jiv-10.1177_08862605261444014 for Evaluating the Effectiveness of an Intimate Partner Violence Training Intervention on Healthcare Providers’ Preparedness, Knowledge, and Experiences: A Mixed-Methods Study From Nepal by Reena Koju, Rachana Shrestha, Jayanti Dhungana, Achyut Lamichhane, Amit Misra, Anna Mia Ekström and Keshab Deuba in Journal of Interpersonal Violence

## References

[bibr1-08862605261444014] Abeid M. Muganyizi P. Mpembeni R. Darj E. Axemo P. (2016). Evaluation of a training program for health care workers to improve the quality of care for rape survivors: A quasi-experimental design study in Morogoro, Tanzania. Global Health Action, 9(1), Article 31735. 10.3402/gha.v9.31735 PMC4951636 27435570

[bibr2-08862605261444014] Alhalal E. (2020a). Nurses’ knowledge, attitudes and preparedness to manage women with intimate partner violence. International Nursing Review, 67(2), 265–274. 10.1111/inr.12584 32301110

[bibr3-08862605261444014] Alhalal E. (2020b). The effects of an intimate partner violence educational intervention on nurses: A quasi-experimental study. Nurse Education in Practice, 47(January), Article 102854. 10.1016/j.nepr.2020.102854 32858301

[bibr4-08862605261444014] Ambikile J. S. Leshabari S. Ohnishi M. (2020). Knowledge, attitude, and preparedness toward IPV care provision among nurses and midwives in Tanzania. Human Resources for Health, 18(1), 1–7. 10.1186/s12960-020-00499-3 32746849 PMC7398074

[bibr5-08862605261444014] Arora S. Rege S. Bhate-Deosthali P. Thwin S. S. Amin A. García-Moreno C. Meyer S. R. (2021). Knowledge, attitudes and practices of health care providers trained in responding to violence against women: A pre- and post-intervention study. BMC Public Health, 21(1), 1–13. 10.1186/s12889-021-12042-7 34724912 PMC8561996

[bibr6-08862605261444014] Braun V. Clarke V. (2006). Using thematic analysis in psychology. Qualitative Research in Psychology, 3(2), 77–101. https://psychology.ukzn.ac.za/?mdocs-file=1176

[bibr7-08862605261444014] British Columbia. (2015). Creating a safety plan–A booklet designed to provide women with strategies to increase their safety in abusive relationships. https://www2.gov.bc.ca/assets/gov/law-crime-and-justice/criminal-justice/victims-of-crime/vs-info-for-professionals/training/creating-safety-plan.pdf

[bibr8-08862605261444014] Clark C. J. Ferguson G. Shrestha B. Shrestha P. N. Batayeh B. Bergenfeld I. Chang S. McGhee S. (2019). Mixed methods assessment of women’s risk of intimate partner violence in Nepal. BMC Women’s Health, 19(1), 1–8. 10.1186/S12905-019-0715-4 30691430 PMC6350343

[bibr9-08862605261444014] Colombini M. Dockerty C. Mayhew S. H. (2017). Barriers and facilitators to integrating health service responses to intimate partner violence in low- and middle-income countries: A comparative health systems and service analysis. Studies in Family Planning, 48(2), 179–200. 10.1111/sifp.12021 28422291 PMC5518204

[bibr10-08862605261444014] Colombini M. Mayhew S. Watts C. (2008). Health-sector responses to intimate partner violence in low- and middle-income settings: A review of current models, challenges and opportunities. Bulletin of the World Health Organization, 86(8), 635–642. 10.2471/BLT.07.045906 18797623 PMC2649453

[bibr11-08862605261444014] Deuba K. Shrestha R. Koju R. Jha V. K. Lamichhane A. Mehra D. Ekström A. M. (2024). Assessing the Nepalese health system’s readiness to manage gender-based violence and deliver psychosocial counselling. Health Policy and Planning, 39(2), 198–212. 10.1093/HEAPOL/CZAE003 38300229 PMC10883662

[bibr12-08862605261444014] Eapen D. J. Santa Maria D. M. (2024). Empowering survivors of intimate partner violence: The role of advanced practice nurses. Journal for Nurse Practitioners, 20(2), Article 104911. 10.1016/j.nurpra.2023.104911

[bibr13-08862605261444014] Etherington N. Baker L. Ham M. Glasbeek D. (2021). Evaluating the effectiveness of online training for a comprehensive violence against women program: A pilot study. Journal of Interpersonal Violence, 36(1–2), 160–183. 10.1177/0886260517725734 29294884

[bibr14-08862605261444014] FORGE. (2013). Safety planning: A guide for transgender and gender non-confirming individuals who are experiencing intimate partner violence. Milwaukee. https://www.forge-forward.org

[bibr15-08862605261444014] Gliner J. A. Morgan G. A. Leech N. L. (2017). Research methods in applied settings (3rd ed.). Routledge.

[bibr16-08862605261444014] Goodman L. Dutton M. A. Weinfurt K. Cook S. (2003). The intimate partner violence strategies index. Development and application. Violence Against Women, 9(2), 163–186. 10.1177/1077801202239004

[bibr17-08862605261444014] Guruge S. Illesinghe V. Gunawardena N. (2021). Healthcare provider responses and preparedness towards caring for females who have experienced intimate partner violence in Sri Lanka. OUSL Journal, 16(2), 65–85. 10.4038/ouslj.v16i2.7507

[bibr18-08862605261444014] Kalra N. Hooker L. Reisenhofer S. Di Tanna G. L. Garcia-Moreno C. (2021). Training healthcare providers to respond to intimate partner violence against women. Cochrane Database of Systematic Reviews, 5, Article CD012423. 10.1002/14651858.CD012423.pub2 PMC8166264 34057734

[bibr19-08862605261444014] Kaplan S. Komurcu N. (2017). Evaluation of effectiveness of health services training given with different methods in combating of intimate partner violence against women: A pilot study. Journal of Family Violence, 32(1), 69–77. 10.1007/s10896-016-9834-y

[bibr20-08862605261444014] Martin-Engel L. Allen J. Alencar A. Levin S. Udezi V. O. Pagels P. Eary R. L. (2021). Improving readiness to manage intimate partner violence in family medicine clinics by collaboration with a community organization. PRiMER, 5, 1–8. 10.22454/primer.2021.717020 34286223 PMC8284494

[bibr21-08862605261444014] McAndrew M. Pierre G. C. Kojanis L. C. (2014). Effectiveness of an online tutorial on intimate partner violence for dental students: A pilot study. Journal of Dental Education, 78(8), 1176–1181. 10.1002/j.0022-0337.2014.78.8.tb05789.x 25086151

[bibr22-08862605261444014] Ministry of Health and Population. (2015). A clinical protocol on gender based violence. https://nepal.unfpa.org/en/publications/clinical-protocol-gender-based-violence

[bibr23-08862605261444014] Ministry of Health and Population, & New Era. (2022a). Nepal Demographic and Health Survey. https://www.dhsprogram.com/pubs/pdf/FR379/FR379.pdf

[bibr24-08862605261444014] Ministry of Health and Population, & New Era. (2022b). Nepal Health facility Survey 2021 Final Report. https://www.dhsprogram.com/pubs/pdf/SPA35/SPA35.pdf

[bibr25-08862605261444014] Mostafa Arrab M. Shabaan Ibrahim H . (2018). Effect of educational training intervention on overcoming nurses’ barriers to screening intimate partner violence against women in outpatient clinics. American Journal of Nursing Research, 6(4), 198–207. 10.12691/ajnr-6-4-8

[bibr26-08862605261444014] Oram S. Fisher H. L. Minnis H. Seedat S. Walby S. Hegarty K. Rouf K. Angénieux C. Callard F. Chandra P. S. Fazel S. Garcia-Moreno C. Henderson M. Howarth E. MacMillan H. L. Murray L. K. Othman S. Robotham D. Rondon M. B. . . . Howard L. M. (2022). The Lancet Psychiatry Commission on intimate partner violence and mental health: Advancing mental health services, research, and policy. The Lancet. Psychiatry, 9(6), 487–524. 10.1016/S2215-0366(22)00008-6 35569504

[bibr27-08862605261444014] Papadakaki M. Petridou E. Kogevinas M. Lionis C. (2013). Measuring the effectiveness of an intensive IPV training program offered to Greek general practitioners and residents of general practice. BMC Medical Education, 13(1), 46. 10.1186/1472-6920-13-46 23537186 PMC3617069

[bibr28-08862605261444014] Pawson R. Tilley N. (1997). Realistic evaluation. Sage.

[bibr29-08862605261444014] Pedersen G. Gebrekristos F. Eloul L. Golden S. Hemmo M. Akhtar A. Schafer A. Kohrt B. (2021). Development of a tool to assess competencies of problem management plus facilitators using observed standardised role plays: The EQUIP competency rating scale for problem management plus. Intervention, 19(1), 107–117. 10.4103/INTV.INTV_40_20

[bibr30-08862605261444014] Potter L. C. Morris R. Hegarty K. García-Moreno C. Feder G. (2021). Categories and health impacts of intimate partner violence in the World Health Organization multi-country study on women’s health and domestic violence. International Journal of Epidemiology, 50(2), 652–662. 10.1093/IJE/DYAA220 33326019

[bibr31-08862605261444014] Rampin R. Rampin V. (2021). Taguette: Open-source qualitative data analysis. Journal of Open Source Software, 6(68), 03522. https://joss.theoj.org/papers/10.21105/joss.03522

[bibr32-08862605261444014] Short L. M. (2006). PREMIS tool-kit instructions. American Journal of Preventive Medicine, 30(2), 180.e1–180.e19.

[bibr33-08862605261444014] Shrestha R. Sapkota D. Mehra D. Ekström A. M. Deuba K. (2023). Feasibility and effectiveness of an intervention to reduce intimate partner violence and psychological distress among women in Nepal: Protocol for the Domestic Violence Intervention (DeVI) cluster-randomized trial. JMIR Research Protocols, 12, Article e45917. 10.2196/45917 PMC10466145 37581909

[bibr34-08862605261444014] Sikder S. S. Ghoshal R. Bhate-Deosthali P. Jaishwal C. Roy N. (2021). Mapping the health systems response to violence against women: Key learnings from five LMIC settings (2015–2020). BMC Women’s Health, 21(1), 1–13. 10.1186/s12905-021-01499-8 34629077 PMC8504083

[bibr35-08862605261444014] Sprague S. (2019). A qualitative evaluation of the implementation of an intimate partner violence education program in fracture clinics. Journal of Family Violence, 34(7), 621–630. 10.1007/s10896-019-00052-4 31929680 PMC6936650

[bibr36-08862605261444014] The EDUCATE Investigators. (2018). Novel educational program improves readiness to manage intimate partner violence within the fracture clinic: A pretest-posttest study. CMAJ Open, 6(4), E628–E636. 10.9778/cmajo.20180150 PMC6298867 30563918

[bibr37-08862605261444014] Walker L. (1979). The battered woman. HarperCollins.

[bibr38-08862605261444014] World Health Organization. (1997). Violence against women health consequences. https://iris.who.int/server/api/core/bitstreams/fbf9389d-2358-4367-ae7a-83894f464ba2/content

[bibr39-08862605261444014] World Health Organization. (2013). Responding to intimate partner violence and sexual violence against women. WHO clinical and policy guidelines. http://apps.who.int/iris/bitstream/10665/88184/1/WHO_RHR_13.10_eng.pdf 24354041

[bibr40-08862605261444014] World Health Organization. (2018). Problem Management Plus (PM+): Individual psychological help for adults impaired by distress in communities exposed to adversity. https://iris.who.int/handle/10665/375604

[bibr41-08862605261444014] World Health Organization, & Pan American Health Organization. (2012). Understanding and addressing violence against women: Intimate partner violence. https://www.paho.org/hq/dmdocuments/2012/paho-violence-women-fs-ipv-2012.pdf

