# Supplementary material for: Evaluating the Effectiveness of an Intimate Partner Violence Training Intervention on Healthcare Providers’ Preparedness, Knowledge, and Experiences: A Mixed-Methods Study From Nepal
Source: J Interpers Violence. 2026 May 19;41(19-20):7949–75. doi: 10.1177/08862605261444014 (PMC13570984; doi:10.1177/08862605261444014)
Supplement: Supplementary file 1 [file sj-docx-1-jiv-10.1177_08862605261444014.docx]

Supplementary Table 1: Pre and post-training changes in perceived preparedness, knowledge, attitudes, and IPV consequences awareness among female HCPs in all districts in Madhesh province, Nepal. Data collected in May-June 2022

|  | **Group 1** | | | | **Group 2** | | | | **Total** | | | |
| --- | --- | --- | --- | --- | --- | --- | --- | --- | --- | --- | --- | --- |
|  | Pretest  (x̄± σ) | Post test  (x̄± σ) | MD  (95% CI) | P value | Pretest  (x̄± σ) | Post test  (x̄± σ) | MD  (95% CI) | P  value | Pretest  (x̄± σ) | Post test  (x̄± σ) | MD  (95% CI) | P value |
| Perceived preparedness | 3.3*  (1.9 – 5.3) | 6.2*  (5.8- 6.8) | 2.7^β^  (1.5 to 3.8) | 0.000^δ^ | 2.6*  (1.6 - 4.1) | 3.9*  (3.2 – 5.7) | 1.2^β^  (0.4 to 2.1) | 0.029 ^δ^ | 3.2*  (1.8- 4.6) | 5.9*  (3.8 - 6.3) | 2.1 ^β^  (1.1 to 2.9) | 0.000 ^δ^ |
| Perceived knowledge | 2.8*  (1.9 – 4.0) | 6.0*  (5.1- 6.5) | 2.9 ^β^  (2.2 to 3.3) | 0.000 ^δ^ | 2.4 ± 0.8 | 4.9 ± 1.3 | 2.4  (1.8 to 3.0) | 0.000 | 2.5*  (1.9 - 3.7) | 5.8*  (4.7 - 6.4) | 2.7 ^β^  (2.0 to 3.1) | 0.000 ^δ^ |
| Actual knowledge | 13.8 ± 4.9 | 18.5 ± 4.2 | 4.7  (3.0 to 6.3) | 0.000 | 13.0 ± 5.2 | 15.0 ± 3.3 | 2  (0.5 to 4.5) | 0.111 | 13.4 ± 5.0 | 16.9 ± 4.2 | 3.4  (1.9 to 4.9) | 0.000 |
| Attitude | 2.5 ± 0.1 | 2.7 ± 0.1 | 0.1  (0.0 to 0.2) | 0.000 | 2.6 ± 0.1 | 2.7 ± 0.2 | 0.1  (0.0 to 0.2) | 0.069 | 2.6 ± 0.2 | 2.7 ± 0.2 | 0.1  (0.0 to 0.2) | 0.000 |
| Consequence of IPV | 6.5 ± 2.2 | 7.7 ± 1.3 | 1.2  (0.2 to 2.2) | 0.019 | 5.2 ± 2.4 | 6.5 ± 0.9 | 1.3  (0.2 to 2.4) | 0.025 | 5.9 ± 2.4 | 7.1 ± 1.2 | 1.2  (0.5 to 2.0) | 0.001 |

Note: x̄ stands for Mean, σ for standard deviation, MD stands for mean difference, and CI stands for confidence interval; ^β^ indicates median difference; *indicates these data did not follow normal distribution, thus median with interquartile range was reported; δ indicates analysis was based on Wilcoxon sign rank test.
